# Comparison of Different Classification Systems for Müllerian Duct Anomalies: A Retrospective Observational MRI Study

**DOI:** 10.3390/medicina62030592

**Published:** 2026-03-21

**Authors:** Laura D’hoore, Eva Decroos, Pieter Julien Luc De Visschere, Ottavia Battaglia, Tjalina Hamerlynck

**Affiliations:** 1 Department of Gynaecology, Ghent University Hospital, 9000 Ghent, Belgium; 2 Department of Human Structure and Repair, Faculty of Medicine and Health Sciences, Ghent University, 9000 Ghent, Belgium; 3 Department of Radiology and Nuclear Medicine, Ghent University Hospital, 9000 Ghent, Belgium; 4 Department of Diagnostic Sciences, Faculty of Medicine and Health Sciences, Ghent University, 9000 Ghent, Belgium

**Keywords:** MRI, Müllerian duct anomalies, classification, interrater reliability

## Abstract

*Background and Objectives*: Müllerian duct anomalies (MDAs) are congenital malformations of the female genital tract for which several classification systems have been proposed. The objective of this study is to estimate the interrater reliability of the American Fertility Society (AFS), European Society of Human Reproduction and Embryology/European Society for Gynaecological Endoscopy (ESHRE/ESGE), American Society for Reproductive Medicine (ASRM) and Congenital Uterine Malformation by Experts (CUME) classification systems for Müllerian duct anomalies. *Materials and Methods*: This retrospective cohort study was conducted at a tertiary care hospital and included 71 patients aged up to 45 years who were assessed for a Müllerian duct anomaly between January 2000 and April 2023. Pelvic MRI images were independently evaluated by four readers, followed by a consensus meeting. The primary outcome was interrater reliability (Krippendorff’s α), and the secondary outcomes were the proportions of indeterminate and unclassifiable cases after consensus meeting. *Results*: The interrater reliability for MDA diagnosis was very low for all the classification systems (AFS α 0.63, 95% CI [0.57, 0.67]; ASRM α 0.46, 95% CI [0.41, 0.52]; ESHRE/ESGE α 0.33, 95% CI [0.29, 0.38]; CUME α 0.57, 95% CI [0.45, 0.72]). After consensus meeting, the ESHRE/ESGE system had more indeterminate cases (9.9%) and the ASRM system had more unclassifiable cases (20.6%). *Conclusions*: All the classification systems for Müllerian duct anomalies had a very low interrater reliability, with more indeterminate cases in the ESHRE/ESGE system and more unclassifiable cases in the ASRM system. We present our recommendations for the improvement of each classification system. The ultimate goal of future research should be the development of a single uniform system integrating the best features of these systems and with clinically relevant cut-off values, considering patients’ reproductive outcomes.

## 1. Introduction

Müllerian duct anomalies (MDAs) are congenital anomalies of the female genital tract that arise from a defect in the formation, canalisation, fusion and/or reabsorption of the Müllerian ducts. The prevalence of MDAs is estimated to be around 5.5%, with a higher prevalence in patients with subfertility and/or a history of repeated miscarriage [1].

For the diagnosis of MDAs, combined laparoscopy and hysteroscopy was long considered the gold standard. However, the invasive nature and the impossibility to take exact measurements has resulted in a shift towards imaging techniques, namely 2D/3D transvaginal ultrasound (TVUS) and magnetic resonance imaging (MRI). Both are high-performing methods, with a high accuracy. To date, MRI is still considered the gold standard technique, though systematic reviews of small studies report promising results for 3D TVUS [2,3].

Currently, there is no universally accepted classification system for MDAs. In 1988, the American Fertility Society (AFS) created the first visual and subjective classification system [4]. In 2013, the European Society of Human Reproduction and Embryology/European Society for Gynaecological Endoscopy (ESHRE/ESGE) developed a classification system evaluating the uterine body, cervix and vagina separately, with measurable definitions of a septate and bicorporeal uterus [5]. In 2016, the American Society for Reproductive Medicine (ASRM, previously AFS) published their measurable definitions of a normal/arcuate, septate and bicornuate uterus (i.e., ‘the morphometric criteria’) [6]. In 2018, Ludwin et al. created the Congenital Uterine Malformation by Experts (CUME) classification of a normal/arcuate versus septate uterus, as studies have described a risk of overdiagnosis of uterine septa using the ESHRE/ESGE classification and a risk of underdiagnosis using the ASRM morphometric criteria [7,8,9,10,11]. In 2021, the ASRM incorporated their measurable criteria in an updated system [12].

The coexistence of different classification systems for MDAs is inconvenient. Ideally, there should be only one universally accepted uniform classification system, integrating the best features of the abovementioned systems. Moreover, an important shortcoming of the existing classification systems is that they are based on expert consensus, without sufficient evidence on reproductive outcomes [13].

To date, no MRI studies have thoroughly explored the reproducibility of all four classification systems for Müllerian duct anomalies. Within this framework, the current study aimed to determine the interrater reliability of the existing AFS, ASRM, ESHRE/ESGE and CUME classification systems based on MRI. Furthermore, the proportions of indeterminate and unclassifiable cases were evaluated. Considering these results, (dis)advantages of each system are described, and recommendations for a future uniform classification system are proposed.

## 2. Materials and Methods

This retrospective cohort study included following patients: aged up to 45 years, with the presence of a uterus, diagnosed and/or treated for an MDA at Ghent University Hospital (Belgium) between January 2000 and April 2023, that signed an informed consent, with a pelvic MRI available and who were not pregnant at the time of the MRI. The quality of the MRI scans was assessed by an expert radiologist and rated as either good or at least suboptimal. Patients who underwent an MRI for other purposes were not included in this study.

Baseline characteristics were defined based on the definitions of The International Glossary on Infertility and Fertility Care [14].

MRI images were independently assessed by four raters, namely a senior expert radiologist in urogenital radiology (P.J.L.D.V.), a senior radiology resident (O.B.) and two senior gynaecology residents (L.D. and E.D.). All were blinded for the clinical findings and for the results of the other raters. All raters received training from a senior urogenital radiologist, which included joint evaluation of MRI images and supervised implementation of the measurement protocol. In addition, a written protocol detailing the standardised MRI measurements was provided.

**Figure 1:**
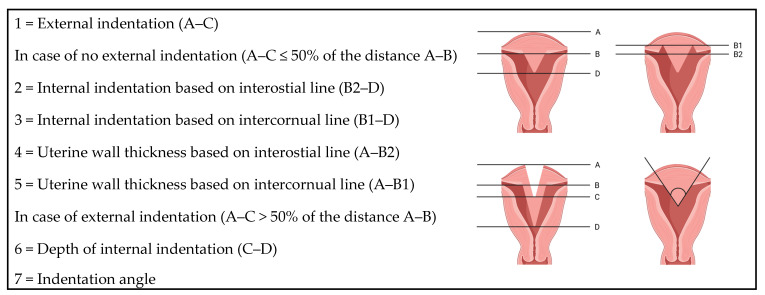
Schematic overview of the standardised measurements. Illustration created with BioRender.com.

Evaluation focused on coronal, axial and axial oblique planes of the T2-weighted images. The T1-weighted images with fat suppression were used to identify haemorrhagic material (e.g., in obstructed anomalies). MDAs were classified according to the AFS, ASRM, ESHRE/ESGE and CUME classification systems using following standardised measurements. A schematic overview is provided in Figure 1.

• Uterine wall thickness (UWT): the distance between the interostial line and a parallel line on the external uterine profile. In case of an external indentation, the distance between the interostial line and the line connecting the external outlines of the two uterine bodies.
• External indentation: the distance between the external outline of the two uterine bodies and the indentation at the fundal midline.
• Internal indentation: the distance between the interostial line and a parallel line on the edge of the indentation at the cavity. In case of an external indentation, the distance between the external indentation at the midline and the edge of the indentation at the cavity.
• Internal indentation angle: angle of the leading edge of the septum.

For the CUME classification, the intercornual line (line connecting the highest point of the endometrial cavity at each side of the uterus) was used instead of the interostial line [3,11,12].

Based on these measurements, all patients were classified according to all four classification systems if applicable. After these primary analyses, a consensus meeting was organised to discuss conflicting classification decisions in order to reach consensus. If consensus could not be reached, a case was labelled ‘indeterminate’. Cases were labelled ‘unclassifiable’ for a certain classification system when the patient’s condition did not fit within the criteria of the classification system. The primary outcome was interrater reliability.

The secondary outcomes were the proportions of indeterminate and unclassifiable cases. Secondary outcomes were compared only between the AFS, ASRM and ESHRE/ESGE classification systems, as the CUME system included only a subpopulation of patients with suspected uterine septum.

Statistical analyses were performed using SPSS (Statistical Package for Social Sciences, version 31, Chicago, IL, USA). Continuous variables were reported as mean ± standard deviation for normally distributed data, and as median with interquartile range for skewed data. Categorical variables were reported as frequencies and percentages. For the interrater reliability, the Krippendorff’s alpha was calculated using the KALPHA macro by Hayes et al. [15] and was interpreted as follows: α < 0.67 very low interrater reliability; 0.67–0.8 low interrater reliability; >0.8 good interrater reliability [16]. To compare the proportion of indeterminate and unclassifiable cases between the different classification systems, a Chi-square/Fisher’s exact test was performed. All statistical analyses were tested two-sided and *p* < 0.05 was considered statistically significant, with Bonferroni correction in case of post hoc comparisons.

## 3. Results

This study included 71 patients, with 29 patients who received their MRI at Ghent University Hospital and 42 patients at other hospitals. Demographic and clinical characteristics are displayed in Table S1.

### 3.1. Interrater Reliability

**Figure 2:**
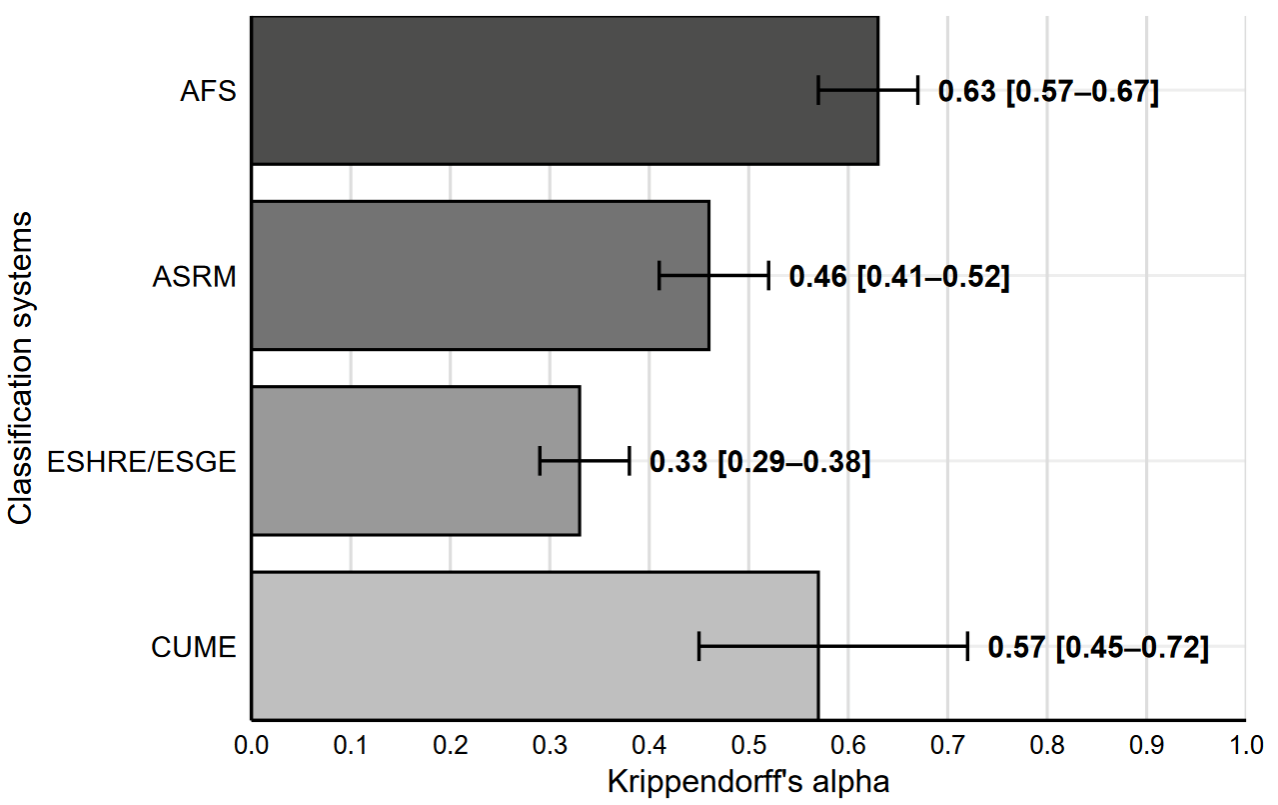
Interrater reliability for the AFS, ASRM, ESHRE/ESGE and CUME classification systems. Data are Krippendorff’s alpha [95% CI].

Figure 2 displays the interrater reliability for the different classification systems, with all systems showing a very low interrater reliability (α < 0.67), although significantly higher for the AFS system compared to the ASRM system, and significantly higher for the ASRM system compared to the ESHRE/ESGE system.

### 3.2. Indeterminate Cases

The proportions of indeterminate cases after consensus meeting according to the different classification systems were as follows (reported as n (%)): AFS 0 (0.0%); ASRM 3 (4.2%), including two uterine and one cervical anomalies; ESHRE/ESGE 7 (9.9%), including three uterine and four cervical anomalies; CUME 0 (0.0%). General Fisher’s exact test indicated a significant difference among the AFS, ASRM and ESHRE/ESGE system (*p* = 0.019), with significantly more indeterminate cases for the ESHRE/ESGE system compared to the AFS system, while other post hoc comparisons were not significant. A supplementary table detailing the indeterminate cases after consensus meeting has been included (Table S2).

### 3.3. Unclassifiable Cases

The proportions of unclassifiable cases after consensus meeting according to the different classification systems were as follows (reported as n (%)): AFS 2 (2.8%); ASRM 14 (20.6%); ESHRE/ESGE (uterine and/or cervical and/or vaginal anomaly) 1 (1.6%); CUME 0 (0.0%). General Chi-square test indicated a significant difference among the AFS, ASRM and ESHRE/ESGE system (*p* < 0.001), with significantly more unclassifiable cases in the ASRM system compared to the AFS and the ESHRE/ESGE system. There was no significant difference between the AFS and ESHRE/ESGE classification system.

## 4. Discussion

In this study, the AFS, ASRM, ESHRE/ESGE and CUME classification systems for Müllerian duct anomalies were evaluated. All systems demonstrated a very low interrater reliability, though the AFS system showed a significantly higher reliability compared to the ASRM system. The ASRM system, in turn, showed a significantly higher reliability than the ESHRE/ESGE system. The ESHRE/ESGE system had significantly more indeterminate cases and the ASRM system had significantly more unclassifiable anomalies after consensus meeting.

### 4.1. Interpretation of Findings

There are several possible explanations for the very low interrater reliabilities and the proportions of indeterminate and unclassifiable cases.

On the one hand, there are some limitations applicable across multiple systems. Firstly, some classifications require quantitative measurements whereby small interindividual and intraindividual measurement variations can change the classification. Specifically, in the ESHRE/ESGE classification, definitions are based on a relative measurement in relation to the UWT, meaning that even submillimetric changes in cases of a very thin UWT can alter the diagnosis. In addition, measuring the internal indentation angle for the ASRM classification was neither reproducible nor practical. Importantly, all these cut-off values are determined based on low-level evidence of reproductive outcomes [5,6,11,17,18]. Hereby, future studies should strive for clinically relevant and easily applicable measurements. Secondly, some classifications are subjective, e.g., a uterus didelphys versus a uterus bicornuate bicollis in the AFS and ASRM classification system. In the ESHRE/ESGE classification, only the category of a complete bicorporeal uterus with double cervix (U3bC2) has been withheld, which should increase interrater reliability. Thirdly, the position of the arcuate uterus is unclear. The AFS classification defined it as a separate category, while the ASRM and CUME considered it as a variant of normal anatomy. ESHRE/ESGE suggested that it could still be associated with impaired reproductive outcomes [4,5,12], classifying it as a type of dysmorphic uterus.

**Figure 3:**
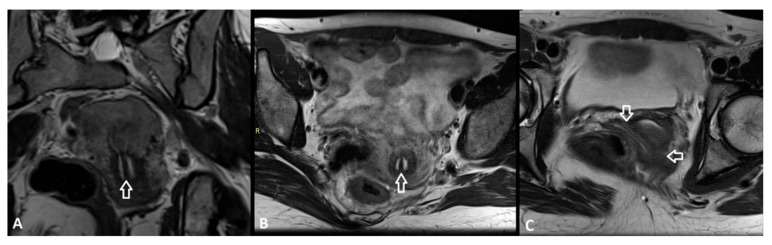
27-year-old female with complete septate uterus, cervix and vagina. Axial T2-weighted MR images at the level of the uterine corpus (**A**), cervix (**B**) and vagina (**C**). The septum is demonstrated with white arrows. The vaginal septum is longitudinal and non-obstructing. This Müllerian duct anomaly is described in the ASRM classification as a ‘complete septate uterus with septate cervix and longitudinal vaginal septum’. In the ESHRE/ESGE classification, this is a U2b C1 V1. The AFS classification only mentions complete septate uterus.

On the other hand, there are some system-specific limitations. Firstly, the AFS system does not classify cervical and/or vaginal anomalies, except for hypoplasia/agenesia. A case demonstrating this limitation is presented in Figure 3.

**Figure 4:**
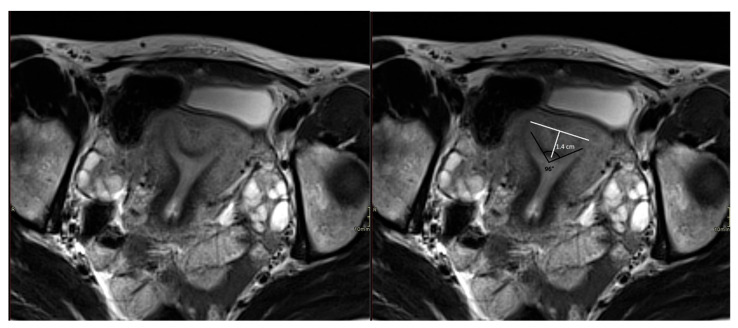
30-year-old female with on ultrasound suspicion of a uterine septum. Axial T2-weighted MR image parallel with the long axis of the uterus. The external uterine contour is convex, and the myometrium is thickened at the level of the fundus, suggestive of a partial uterine septum. The indentation depth is 1.4 cm, and the septum angle is 96°. According to the AFS classification this is a partial septate uterus. In the ESHRE/ESGE classification this is U2a and in CUME the criteria are fulfilled for a septate uterus. In the ASRM classification, however, this Müllerian duct anomaly is indeterminate because the septum has a length of >1 cm and an angle of >90°, excluding the diagnosis of septate uterus (the septum angle should be <90° in that case) as well as normal/arcuate uterus (the septum length should be <1 cm in that case).

Secondly, the ASRM classification has a higher number of unclassifiable cases. In particular, the following common anomalies cannot be classified: (a) patients with a septum > 1 cm but an angle ≥ 90° or with a septum ≤ 1 cm but an angle < 90°, (b) patients with a complete uterine septum, with a normal, double, or septate cervix but without vaginal septum. An example of an unclassifiable case according to the ASRM system is presented in Figure 4. Moreover, some anomalies appear twice in the ASRM classification. They describe that the physician may classify the anomaly as primarily uterine or vaginal, depending on its presentation. This may lower the interrater reliability [12].

Thirdly, the ESHRE/ESGE system has a limitation in classifying cervical anomalies, as it only includes (unilateral/bilateral) cervical aplasia, although hypoplasia was encountered in several cases in our study. Finally, the CUME classification uses the intercornual line instead of the interostial line because of difficulties identifying the tubal ostia and the underestimation of the internal indentation as the interostial line may be more caudal than the intercornual line. In our study, all raters agreed that the difference between the interostial and intercornual line was smaller than the inherent measurement error of MRI.

Apart from the limitations of the classification systems, technical limitations of MRI may also reduce interrater reliability, even though MRI is considered to be highly accurate in the diagnosis of MDAs. For example, interpretation of cervical and vaginal anomalies can be difficult on MRI. Since the AFS classification does not focus on these anomalies, this may explain why the interrater reliability was significantly higher in our study. However, in daily practice, this classification is less useful precisely because it does not include information on cervical and vaginal anomalies.

### 4.2. Comparison with Literature

To our knowledge, only three studies to date have compared the interrater reliability of different MDA classification systems. In 2015, Ludwin et al. [19] estimated the interrater reliability of the AFS classification supplemented with the 2016 ASRM morphometric criteria, and the ESHRE/ESGE classification system. In this study, the 3D ultrasound volumes of 112 patients (50 patients with a congenital uterine anomaly and 62 healthy controls) were evaluated by two expert raters. The interrater reliability of the AFS classification with 2016 morphometric criteria was higher than that of the ESHRE/ESGE classification for uterine anatomy classification (κ 0.96 95% CI 0.85–1.00 and κ 0.80 95% CI 0.65–0.95, resp.). Moreover, it was higher for diagnosing septate uterus (κ 0.96 95% CI 0.78–1.00 and κ 0.76 95% CI 0.57–0.94, resp.) and for distinguishing anomalous from normal uteri (κ 0.94 95% CI 0.75–1.00 and κ 0.77 95% CI 0.58–0.95, resp.). They concluded that the reliability of the ESHRE/ESGE system may be clinically insufficient as the κ values were below clinically relevant cutoffs [19]. These results are difficult to compare with our findings because Ludwin et al. applied the AFS classification with 2016 morphometric criteria and the temporary ESHRE/ESGE recommendations for measuring the UWT in the sagittal plane (considering that the 2016 Thessaloniki ESHRE/ESGE consensus guidelines had not yet been developed) [20]. In contrast, we used the AFS classification and the most recent 2021 ASRM classification. Our measurements of the UWT followed the 2016 Thessaloniki ESHRE/ESGE consensus guidelines, using the midcoronal plane of the uterus.

In 2021, Peixoto et al. [21] evaluated the interrater reliability among non-expert raters for the classification of a septate uterus using the ASRM, ESHRE/ESGE, and CUME classification systems. The CUME definition was applied using all three criteria (internal indentation angle < 140°, internal indentation: UWT > 110%, and internal indentation ≥ 10 mm). A total of 47 3D volumes of women with suspected uterine anomalies were assessed. The interrater reliability was very good for the ASRM and CUME (κ 0.96; 95% CI 0.88–1.00; and κ 0.91; 95% CI 0.79–1.00, resp.) and good for the ESHRE/ESGE classification system (κ 0.74; 95% CI 0.55–0.92). Notably, uteri were classified as septate only when both ASRM criteria or at least two CUME criteria were met. Otherwise, they were classified as normal. As a result, no unclassifiable cases remained [21].

In the study by Elshetry et al. (2024) [22], 76 MRI examinations were assessed by four radiologists using the ASRM and ESHRE/ESGE classification systems. Overall interrater reliability was moderate for both systems (κ 0.599 vs. κ 0.429, resp.), but significantly better for ASRM. An important remark is that some pairwise interrater reliabilities were higher for both systems compared to those observed among all four raters. Notably, the proportion of unclassifiable cases based on the ASRM classification ranged from 15.8% to 26.3% across the four readers, whereas no cases were unclassifiable using the ESHRE/ESGE system [22].

In addition, Al Najar et al. (2022) [23] compared the AFS, the ESHRE/ESGE and the ASRM classification system on MRI in a descriptive study without calculation of interrater reliability. Although they refer to the ASRM classification as simpler and more user-friendly, they also described a high rate of unclassifiable cases [23].

It is possible that the higher interrater reliabilities reported in other studies can be explained by differences in the group of raters (all experts vs. all non-experts vs. mixed groups) and the number of raters. In addition, different diagnostic techniques were used (3D ultrasound vs. MRI).

### 4.3. Strengths and Limitations

The strength of our study is the evaluation by four independent raters. The inclusion of both radiologists and gynaecologists on the one hand, and both MRI experts and non-experts on the other hand, increased the generalisability of our results, as this resembles daily practice. Moreover, four different classification systems were evaluated (AFS, ASRM, ESHRE/ESGE, and CUME) and a consensus meeting was organised to discuss all conflicting classification decisions. In addition, MRI was utilised as the diagnostic test, which is still considered the gold standard for diagnosing Müllerian duct anomalies.

This study also has some limitations. Firstly, intrarater reliability was not evaluated, which may limit the interpretability of the reliability estimates. Nevertheless, we made efforts to enhance the consistency of individual raters’ measurements through targeted training and the implementation of a written protocol.

This study regarded MRI performed in different hospitals over a longer period of time, resulting in different MRI scanning protocols, although the most relevant scan sequences (coronal T2, axial T2, axial oblique T2 and axial T1 with fat suppression) were present in all the exams. Moreover, only good or at minimum suboptimal MRI images were included. On the other hand, incorporating MRI scans from multiple centres, with varying protocols, reflects real clinical practice. The retrospective nature of the study had minimal additional impact, as no clinical data were necessary for the review of the MRI images.

More recently, it has been described that 3D TVUS may be a valuable alternative to MRI in the diagnosis of MDAs, as a less expensive and more available method [2]. However, 3D TVUS was not available for all patients retrospectively included in this study.

Furthermore, not all anomalies are equally represented in our study; therefore, the performance of the classification systems for some rare anomalies might not be representative. In addition, MRI was performed mostly to elaborate on uterine anomalies, which were therefore overrepresented in our study sample compared to cervical and vaginal anomalies. This could introduce selection bias.

Importantly, the major strength of this study is its exploration of why interrater reliabilities are low and the proportions of indeterminate and unclassifiable cases are high. The contributing authors provide a thorough description of the limitations of the different classification systems and aim to contribute to the refinement of these systems in the future.

### 4.4. Recommendations

Based on the findings of our study, several recommendations can be made. Firstly, as quantitative measurements can have a significant impact on the intrarater and interrater reliability, they should be simple and reproducible. The use of multiple measurements (e.g., to calculate the ratio of internal indentation to UWT) for the classification of an anomaly should be avoided, as this increases the measurement error and is more time-consuming and challenging in everyday practice. Therefore, we suggest an absolute measurement of the length of the external and internal indentation without consideration of the uterine wall thickness or the indentation angle. Additionally, since the current cut-off values in all classification systems are based on limited evidence, future research should take into account the reproductive outcomes to determine clinically relevant cut-off values.

Secondly, redundant categories should be removed, e.g., the category ‘uterus didelphys’ should no longer be withheld and could be replaced by a ‘bicornuate uterus with double cervix’, since the distinction is rather subjective. In the ASRM classification, anomalies that appear twice should be listed only once.

Thirdly, the proportion of unclassifiable cases should be reduced or ideally even completely avoided, e.g., we suggest adding ‘hypoplasia’ to the categories C3 ‘unilateral cervical aplasia’ and C4 ‘cervical aplasia’ in the ESHRE/ESGE system.

We suggest that a single comprehensive uniform MDA classification system should be developed, integrating the best features of all classification systems. In our opinion, this new classification system should provide a practical approach for classifying uterine, cervical, and vaginal anomalies, ensuring a high interrater reliability and a low rate of unclassifiable cases, with clinically relevant cut-off values, considering patients’ reproductive outcomes. An overview of these suggestions is provided in Table 1.

## 5. Conclusions

This study evaluated the AFS, ASRM, ESHRE/ESGE and CUME classification systems for Müllerian duct anomalies. All the classification systems showed a very low interrater reliability with more indeterminate cases according to the ESHRE/ESGE system and more unclassifiable cases according to the ASRM system. Based on these findings, recommendations are proposed for improvement of the classification systems.

This study hereby provides clinically relevant insights into the performance and limitations of existing classification systems, highlighting the need for and guiding evidence-based refinement. The ultimate goal of future research should be the development of a single uniform system integrating the best features of these systems and with clinically relevant cut-off values, considering patients’ reproductive outcomes.

## Figures and Tables

**Table 1:** Suggestions for improvement of the existing classification systems for Müllerian duct anomalies. ^1^ UWT = uterine wall thickness.

Aims	AFS	ASRM	ESHRE/ESGE	CUME
1. Simplify quantitative measurements		Definitions based on an absolute measurement easy to perform in daily practice AND relevant for reproductive outcomes Consensus on use of the interostial versus intercornual line should be reached		
		Use septum length, no use of septum angle to define septate uterus	Use septum length, no use of UWT ^1^ to define bicornuate and septate uterus	/
2. Remove redundant categories	Keep ‘bicornuate uterus with double cervix’, remove ‘uterus didelphys’			
		List anomalies only once		
	More research on the position of ‘arcuate uterus’			
3. Design a comprehensive classification system (to reduce unclassifiable cases)		Add ‘complete uterine septum, with a normal, double or septate cervix, without a vaginal septum’	Add ‘hypoplasia’ to ‘unilateral cervical aplasia’ (C3) and ‘cervical aplasia’ (C4)	
Combine clinical examination, ultrasound, MRI and intraoperative findings				

## Data Availability

The raw data supporting the conclusions of this article will be made available by the authors on request.

## Supplementary Materials

The following supporting information can be downloaded at: https://www.mdpi.com/article/10.3390/medicina62030592/s1, Table S1: Demographic and clinical characteristics, Table S2: Indeterminate cases.

## Abbreviations

The following abbreviations are used in this manuscript:

AFS	American Fertility Society
ESHRE/ESGE	European Society of Human Reproduction and Embryology/European Society for Gynaecological Endoscopy
ASRM	American Society for Reproductive Medicine
CI	Confidence Interval
CUME	Congenital Uterine Malformation by Experts
MDA (MDAs)	Müllerian duct anomaly (Müllerian duct anomalies)
MRI	Magnetic resonance imaging
2D	Two-dimensional
3D	Three-dimensional
TVUS	Transvaginal ultrasound
UWT	Uterine wall thickness
SPSS	Statistical Package for Social Sciences
