# Supplementary material for: Comparison of Different Classification Systems for Müllerian Duct Anomalies: A Retrospective Observational MRI Study
Source: Medicina (Kaunas). 2026 Mar 21;62(3):592. doi: 10.3390/medicina62030592 (PMC13028389; doi:10.3390/medicina62030592)
Supplement: Supplementary file 1 [file medicina-62-00592-s001.zip › medicina-3847283-supplementary.pdf]

Table S1. Demographic and clinical characteristics

	N (missing)	Descriptive statistic[a]
**Age at time of MRI (years)**	71 (0)	25.6 ± 6.53
**Previous pregnancy[b]**	65 (6)	18 (27.7%)
**Previous live birth[b]**	65 (6)	5 (7.7%)
**Previous Caesarean section[b]**	66 (5)	2 (3.0%)

[a] Data are mean ± standard deviation or n (%).

[b] Obstetric history includes all events prior to the MRI. The following definitions were used: previous pregnancy was defined as one or more gestational sacs or definitive clinical signs of pregnancy diagnosed by ultrasonographic visualisation. In addition to intrauterine pregnancy, it includes a clinically documented ectopic pregnancy. Previous live birth was defined as a delivery after 22 completed weeks of gestational age, showing any evidence of life [14].

Table S2. Indeterminate cases

Case number	Classification system with discrepancy	Individual classification			
		Rater 1	Rater 2	Rater 3	Rater 4
3	ESHRE/ESGE (uterine)	U0	U2a	U0	U2a
14	ESHRE/ESGE (cervical)	Unclassifiable	C2	C1	C0
21	ESHRE/ESGE (cervical)	C2	C2	C1	C1
27	ESHRE/ESGE (cervical)	Unclassifiable	C0	C0	C0
	ESHRE/ESGE (uterine)	U0	U0	U2a	U2a
32	ASRM	Complete septate uterus with duplicated cervices and longitudinal vaginal septum	Complete septate uterus with duplicated cervices and longitudinal vaginal septum	Complete septate uterus with septate cervix and longitudinal vaginal septum	Unclassifiable
	ESHRE/ESGE (cervical)	C2	C2	C1	C1
36	ESHRE/ESGE (uterine)	U0	U2a	U2a	U2a
42	ASRM	Partial septate uterus	Unclassifiable	Partial septate uterus	Partial septate uterus
43	ASRM	Partial septate uterus	Unclassifiable	Combined bicornuate septate uterus	Unclassifiable
